# Longitudinal Changes in the Adjusted Body Mass Index (BMI) Percentile Among Children in Riyadh, Saudi Arabia, During and After the COVID-19 Lockdown

**DOI:** 10.3390/healthcare13222875

**Published:** 2025-11-12

**Authors:** Amal Alhakami, Ebtihag O. Alenzi, Najla Ali Algariri, Rawan Abdulaziz Assiri, Hala Muidh Alqahtani

**Affiliations:** 1 Department of Pediatrics, College of Medicine, Princess Nourah bint Abdulrahman University, P.O. Box 84428, Riyadh 11671, Saudi Arabia; 2 Family and Community Medicine Department, College of Medicine, Princess Nourah bint Abdulrahman University, P.O. Box 84428, Riyadh 11671, Saudi Arabia; 3 Pediatrics Department, King Abdullah bin Abdulaziz University Hospital, P.O. Box 84428, Riyadh 11671, Saudi Arabia; najlaalgariri@gmail.com (N.A.A.); rassiri@kfmc.med.sa (R.A.A.); halaalessa0@gmail.com (H.M.A.)

**Keywords:** obesity, overweight, adolescent, child, BMI, COVID-19

## Abstract

**Background:** The COVID-19 pandemic has imposed significant changes on daily life. It negatively impacts children’s physical activity and lifestyle behaviors, which may cause accelerated weight gain during the COVID-19 pandemic. This study aims to evaluate children’s longitudinal age- and sex-adjusted body mass index (BMI) percentile changes during and after the COVID-19 pandemic. **Methods:** Height and weight data were obtained from electronic medical records for children (ages 2–18) visiting outpatient clinics during three periods: pre-COVID-19 lockdown, during COVID-19 lockdown, and post-COVID-19 lockdown. According to the availability of anthropometric information, three partially overlapping cohorts were formed: Cohort 1 (N = 934, pre- and during lockdown), Cohort 2 (N = 1129, during and post-lockdown), and Cohort 3 (N = 203, data from all three periods). **Results:** During the lockdown, the mean changes in percentiles of age- and sex-adjusted BMI were 6% ± 0.23, while after the lockdown, there were smaller mean changes in BMI percentiles (1% ± 0.19). There were significant associations of age and the baseline BMI categories with the change in the means of the adjusted BMI percentile of children during and after the COVID-19 pandemic (*p*-value < 0.001). In addition, there were significant associations of gender and the baseline BMI categories with the change in the means of the adjusted BMI percentile of children over a three-time series (*p*-value: <0.04, <0.001, respectively). **Conclusions:** In conclusion, children experienced increased BMI during and after the COVID-19 lockdown. This result highlights the importance of structured interventions to mitigate the consequences during challenging times on children’s health.

## 1. Introduction

Coronavirus disease 2019 (COVID-19), caused by severe acute respiratory syndrome coronavirus 2 (SARS-CoV-2), a novel coronavirus first identified as an epidemic in Wuhan, China, in December 2019 [1]. It has spread worldwide and was declared by the World Health Organization as a pandemic in March 2020 [2]. During the COVID-19 pandemic, several unprecedented measures were implemented worldwide to curb the spread of the infection [3]. Due to the lack of effective therapeutic agents in the initial pandemic stage, several nonpharmacological measures were taken to control the spread of infection, such as social distancing and lockdown [3]. In Saudi Arabia, the lockdown was first enforced on 25 March 2020, to bring the COVID-19 spread under control [4].

Children’s school attendance, participation in recreational activities, and structured daily mealtimes are essential in controlling child obesogenic behavior [5,6]. RC. Whooten et al. reported that children with a three-day/week school physical activity program had improvements in BMI compared to a two-day/week program [7]. Moreover, it was found that children are at increased risk of weight gain during summer vacation due to more remarkable unhealthy behavioral changes, such as a sedentary lifestyle, changes in sleep timing, and an increase in screen time [8].

The implemented restrictions during the COVID-19 Lockdown (e.g., closed schools, and social isolation) negatively impacted children’s mental and social health, physical activity, and lifestyle behaviors [9,10,11]. Interventions to control the COVID-19 pandemic have resulted in a significant increase in screen time and less participation in physical activity [12,13]. Besides these factors, children during the COVID-19 pandemic had increased engagement in unhealthy eating behaviors and the consumption of discretionary meals, such as potato chips and sweetened beverages [13], all of which contributed to accelerated weight gain and worsening childhood obesity worldwide.

Several studies have evaluated children’s weight status during the COVID-19 pandemic [11]. A study conducted in the US among participants aged 2–19 years, utilizing an electronic medical records database, reported an increase in children’s BMI during the COVID-19 pandemic period [14]. A study involving 1770 children with a mean age of 8.7 years reported an accelerated change in BMI z-score of +0.31 during the COVID-19 pandemic compared to before the pandemic [15]. Moreover, a study conducted in Turkey involved data from 8871 students measured in March 2022, which reported an increase in the prevalence of obesity compared to the 2005, 2009, and 2014 studies [16]. Additionally, a study that included data from children across China reported a significant increase in obesity prevalence of 1.86% during the COVID-19 lockdown [17].

Changes in weight gain during the COVID-19 lockdown showed variability according to age, gender, and baseline weight status. High BMI z-score increases were observed among children aged 7–11 years and adolescents in the first half of 2020, compared with 2019 [17]. A retrospective study in the US reported that Children’s ∆BMI-adjusted (ΔBMIadj) accelerated growth was more evident in ages 8–12. [18] Girls experienced an acceleration in z-BMI change of +0.33 compared to +0.29 in boys during the pandemic, compared to previous years [15]. In addition, children with the highest baseline ΔBMIadj had the most significant increases in ΔBMIadj above baseline during the pandemic [18]. In contrast to this finding, another study reported that children who were overweight or obese before COVID-19 had not experienced weight gain during the pandemic [15].

While previous studies have explored changes in BMI among children in different populations during the COVID-19 pandemic, studies on Saudi Arabia are scarce. In addition, studies evaluating the influence of the COVID-19 pandemic on children’s weight are limited. Weight gain in children during the COVID-19 pandemic could be challenging to reverse and has long-term untoward effects on a child’s health [19,20]. These effects may remain after restrictions have been lifted, despite the return to usual daily activities [21,22]. Therefore, this study aims to evaluate the long-term changes in age- and sex-adjusted body mass index (BMI) percentile among children during and after the COVID-19 pandemic lockdown. Furthermore, the study used electronic clinical data from a university hospital in Riyadh, Saudi Arabia, which provides objective measurement rather than self-reported data. In addition, this study will give region-specific evidence to help health authorities implement strategies to mitigate obesity in children during changing times, such as the COVID-19 pandemic.

## 2. Methods

### 2.1. Study Design

This is a retrospective cohort study using a convenience sample technique of pre-existing data extracted from patients’ electronic health records at King Abdullah bin Abdulaziz University Hospital (KAAUH), Riyadh, Saudi Arabia. Children’s height and weight are routinely taken during clinic visits and were retrieved from electronic health records. For this study, we divided the data into three periods: pre-, during, and after the COVID-19 lockdown, to identify children’s weight changes. The pre-COVID-19 lockdown period includes data from September 2019 to February 2020. The COVID-19 lockdown, or “pandemic period,” covers data from September 2020 to September 2021, which is six months after the pandemic began. The after-COVID-19 lockdown period includes data from July 2022 to December 2022, after six months of returning to school and normal daily activities. The study’s main goal is to assess children’s longitudinal age- and sex-adjusted BMI percentile changes during and after the coronavirus pandemic.

### 2.2. Study Population and Covariates

All individuals aged 2–18 years who visited pediatric outpatient clinics at KAAUH during the defined time periods and had complete anthropometric data (height and weight) available in their records were eligible for inclusion. Based on the availability of anthropometric measurements in medical records, the data were categorized into three retrospective cohorts or samples:

1. Cohort 1 (N = 934): Children who had anthropometric measurements recorded in both before and during the COVID-19 lockdown. This sample was used to evaluate the longitudinal adjusted BMI percentile change during the COVID-19 pandemic lockdown.
2. Cohort 2 (N = 1129): Children with anthropometric measurements recorded during the COVID-19 pandemic lockdown and following the lockdown. This sample was used to evaluate longitudinal age- and sex-adjusted BMI percentile changes within individuals from during to after the lockdown.
3. Cohort 3 (N = 203): Children with anthropometric measurements recorded in all three periods: pre-COVID-19 pandemic, during the lockdown, and after the lockdown. This sample was used to evaluate within-subject changes across the entire pandemic timeline.

Since this was a retrospective study using previously recorded clinical data, no direct recruitment or informed consent was required. Children were selected based on their availability in the existing records. To protect patient confidentiality, all identifying information was removed during data extraction. A random identification number was assigned to each case to enable linking of anthropometric data over time for longitudinal analysis.

Demographic and anthropometric measurements were extracted from medical records. Demographic variables consisted of age and gender. Anthropometric measurements included height and weight, which were used to calculate body Mass Index (BMI; kg/m^2^). The age and sex-adjusted BMI percentiles were determined based on WHO’s child growth standards [23]. Based on Centers for Disease Control and Prevention (CDC) Atlanta, GA, USA guidelines [24], the adjusted BMI percentiles for age and gender were categorized into underweight (less than 5%), healthy weight (from 5% to less than 85%), overweight (from 85% to less than 95%), and obese (more than or equal to 95%).

### 2.3. Sample Size

The sample size was calculated based on the assumptions of a 25.7% prevalence of obesity among children in Saudi Arabia [25], and a 5% margin of error. The required sample size is estimated to be at least 294 children.

### 2.4. Statistical Analyses

Descriptive statistics were presented as frequencies with percentages (%) for categorical data and means with standard deviations (SDs) for continuous data. The normality of the main outcome and continuous variable “change in the adjusted BMI percentile) was evaluated using values of skewness and kurtosis, and by assessing the visual inspection of histograms. The univariate associations of the outcome (change in the adjusted BMI percentile) and the independent variables (Age group, gender, and the baseline BMI categories) were examined using independent *t*-tests for two groups and one-way ANOVA for more than two groups. Using cohort 3, the repeated-measures analysis of variance (ANOVA) was used to assess if there is a significant change in the children’s adjusted BMI percentile over three time series (before COVID-19 lockdown, during COVID-19 lockdown, after COVID-19 lockdown). Any test with a *p*-value less than or equal to 0.05 was considered significant. All statistical analyses were conducted using IBM Statistical Package for the Social Sciences (SPSS) software version 25.

## 3. Results

**Figure 1:**
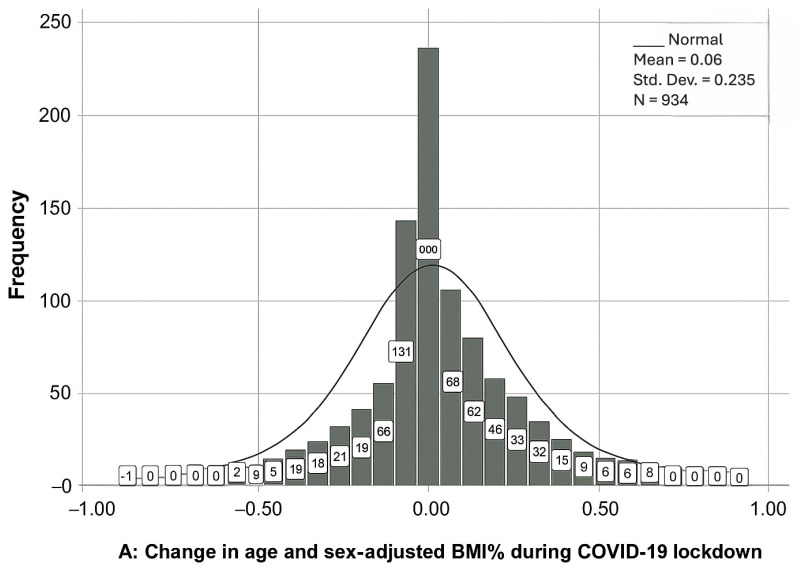

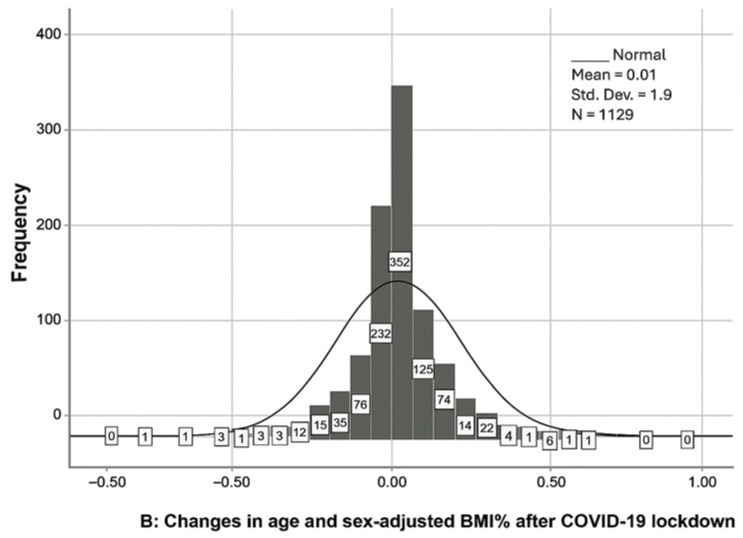
Distribution of changes in the adjusted BMI percentile in cohort 1 (**A**) and cohort 2 (**B**).

The distribution of changes in percentiles of age- and sex-adjusted BMI in children during the COVID-19 lockdown was normal, as it is presented in Figure 1A. The change was 6% ± 0.23 with kurtosis = 1.9 and skewness 0.07. The main characteristics of cohort 1 were displayed in Table 1. About 54% of children were males and 46% were females. Half of the children were 7 to 12 years old, 45% were older than 12, and about 5% were 6 or younger. Most children (60%) had a healthy weight before the COVID-19 lockdown.

The univariate associations of the change in the adjusted-BMI percentile with gender, age, and the baseline BMI categories (before COVID-19 lockdown) were demonstrated in (Table 1). The association of the change in the adjusted-BMI percentile during the COVID-19 lockdown with gender was not statistically significant. However, age groups and the baseline BMI categories were significantly associated with the change in the adjusted BMI percentile. Children whose ages were 6 years or younger had a 13% decrease in their adjusted BMI percentile. In comparison, those whose ages were from 7 to 12 years old and those whose ages were older than 12 years old had 8% and 5% increases in their adjusted BMI percentile during the COVID-19 lockdown, respectively. Being underweight and having a healthy weight before the COVID-19 lockdown were associated with a 13% and 8% increase in the adjusted BMI percentile during the COVID-19 lockdown, respectively. On the other hand, being overweight and obese before the COVID-19 lockdown was associated with a 5% and 4% decrease in the adjusted BMI percentile during the COVID-19 lockdown, respectively.

The distribution of changes in adjusted-BMI percentile in children after COVID-19 lockdown was presented in (Figure 1B). The mean of change was 1% ± 0.19 with kurtosis = 5.77 and skewness—0.24. The description of cohort 2 and the associations of the sample’s characteristics with age and sex-adjusted-BMI percentile after COVID-19 lockdown were displayed in (Table 2). About 52% of children were males and 48% were females. Forty one percent of the children’s ages were less than or equal to 6 years old, 39% were from 7 to 12 years old, 21% of ages were older than 12 years old. About 57% had healthy weight during COVID-19 lockdown.

Likewise, in cohort 1, the association of the change in the adjusted-BMI percentile after the COVID-19 lockdown with gender was not statistically significant in cohort 2. However, age group and the baseline BMI categories (during the COVID-19 lockdown) were significantly associated with the change in the adjusted-BMI percentile after the COVID-19 lockdown. Ages of 6 years or younger were significantly associated with a 2% decrease in the adjusted-BMI percentile, while ages from 7 to 12 years old and ages older than 12 years old had 3% and 2% increase in the adjusted-BMI percentile after the COVID-19 lockdown, respectively. Being underweight and healthy weight during the COVID-19 lockdown was associated with an 8% and 2% increase in the adjusted-BMI percentile after the COVID-19 lockdown, respectively. On the other hand, being overweight and obese during the COVID-19 lockdown was associated with a 5% and 4% decrease in the adjusted-BMI percentile after COVID-19 lockdown, respectively.

**Figure 2:**
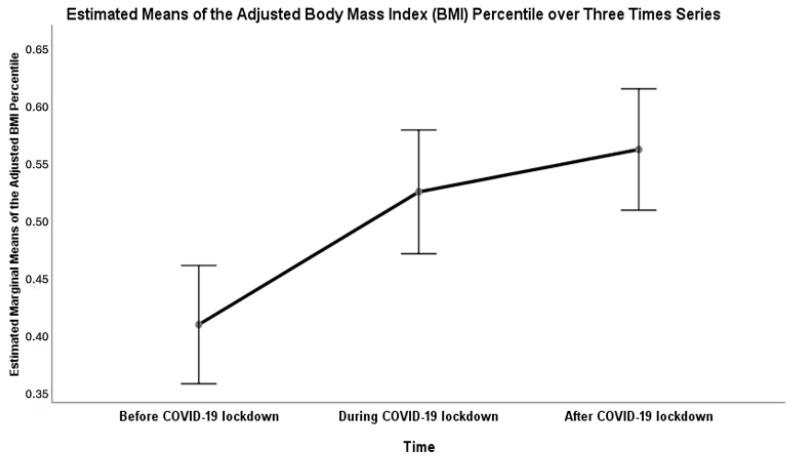
The means of the adjusted BMI percentile among children over three time series using cohort 3.

**Figure 3:**
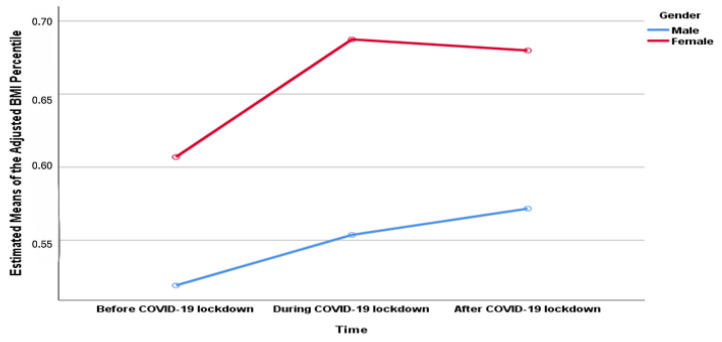

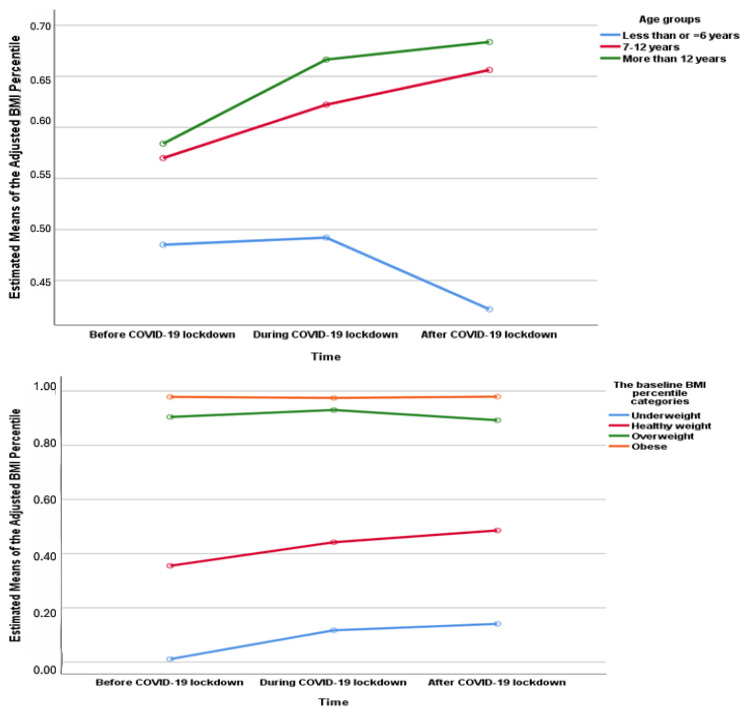
Repeated measures for means of the adjusted BMI percentile over three time series for three factors: Gender; Age groups; The baseline Body Mass Index (BMI) Categories.

For cohort 3, the results of repeated-measures ANOVA showed a significant (Wilk’s lambda = 0.763, F (2, 201) = 31.23, *p* < 0.001, η^2^ = 0.237) effect of time on the children’s adjusted-BMI percentile over the three time series: before COVID-19 lockdown, during COVID-19 lockdown, and after COVID-19 lockdown (Figure 2). Also, changes in the means of the adjusted BMI percentile over a three-year series were displayed based on three factors: gender, age groups, and the baseline BMI categories before the COVID-19 lockdown (Figure 3). There were significant univariate associations of gender and the baseline BMI categories with the change in the means of the adjusted-BMI percentile of children over three time series (Table 3).

## 4. Discussion

Our findings revealed interesting patterns in BMI changes during the three phases of the COVID-19 pandemic. During the lockdown, we observed a normal distribution of changes in BMI percentiles, with a mean change of 6% ± 0.23. This suggests that, on average, children experienced an increase in BMI during this period. These results were consistent with previous findings of a systematic review and meta-analysis, which showed a significant body weight gain among children during the COVID-19 lockdown [11]. However, it is important to note that these changes varied based on age and baseline BMI categories.

In our study, the main increase in BMI percentiles was among school-aged children (7–12 years). Children aged six years or younger demonstrated a significant decrease in their adjusted BMI percentiles, while those aged 7–12 years and older than 12 years showed an increase in their adjusted BMI percentiles. Moreover, the baseline BMI categories before the lockdown played a pivotal role in the changes observed. Children who were underweight or had a healthy weight before the lockdown experienced increases in their adjusted BMI percentiles, whereas those who were overweight or obese showed decreases in their adjusted BMIs. Supporting our findings, data from China indicated that school-aged children experienced the most significant BMI increase during the pandemic [26].

The increase in childhood obesity in our study in children aged more than 7 years was consistent with the findings in a large study conducted in the USA, which found that children whose ages were from 5 to 11-year-olds showed a 157 times increase in BMI, compared with 0.91 among older children, with an increase in the prevalence of overweight/obesity from 36.2% to 45.7% in this age group during the pandemic [27].

In contrast to our finding, a study from England showed that the prevalence of obesity increased among children aged four to five years by 9.9% in 2019–2020 and by 14.4% in 2020–2021, but decreased in 2021–2022 [28].

According to the baseline weight status, the increase in weight was more evident in children with a baseline underweight or a healthy weight. This is opposite to other studies that showed weight gain is observed in children with preexisting obesity [29,30]. Several studies have shown that baseline overweight and obesity were associated with a significant increase in children’s BMI during the COVID-19 pandemic [14,31,32]. Similar to our finding, children with normal weight before the pandemic were about eight times more likely to be obese during the pandemic [15]. These findings highlight the potential impact of pre-existing weight status on BMI changes during periods of restricted physical activity and changes in routine.

After the lockdown, we observed a smaller mean change in BMI percentiles (1% ± 0.19). This suggests stabilization or regression to pre-lockdown BMI levels. Similar to the findings during the lockdown, age and baseline BMI categories were significantly associated with changes in BMI percentiles after the lockdown. Children aged six years or younger had a decrease in BMI percentiles, while those aged 7–12 years and older than 12 years showed an increase in their adjusted BMIs. Additionally, the baseline BMI categories during the lockdown were associated with changes in BMI percentiles after the lockdown. Children who were underweight or had a healthy weight during the lockdown experienced increases in their adjusted BMI percentiles. At the same time, those who were overweight or obese showed decreases in their adjusted BMIs.

These findings indicate that the effects of the COVID-19 lockdown on the adjusted BMI percentiles of children may have non-fixed patterns across age groups and baseline weight status. The observed increases in BMI percentiles during and after the lockdown period among children aged 7 years and older, as well as those with a baseline underweight or healthy weight status, may be partly explained by behavioral or developmental factors. Consistent with Italian and Canadian studies, older children generally have more autonomy over their dietary choices and activity levels, which during lockdown may have led to greater exposure to unhealthy food, reduced physical activity, increased screen time, and disrupted sleep routines [12,33]. Additionally, children with underweight or healthy weight may also receive less parental or clinical attention regarding weight management, unlike those classified as overweight or obese, as documented in a previous European study [31].

Furthermore, the significant effect of time on BMI percentiles over the three-time series (before, during, and after the lockdown) suggests that the pandemic and associated restrictions had a short-term negative impact on children’s BMI. A study conducted in Australia on children aged ≤18 years showed that BMI and weight have returned to pre-pandemic rates despite the initial increases immediately following COVID-19 restrictions [34]. A study examining the BMI trend after the lifting of lockdown restrictions has shown that children with baseline obesity have continued to gain weight post-pandemic [21]. In addition, a study from Argentina assessing the long-term effect of lockdown on children aged 6–9 has shown that boys, unlike girls, have continued to gain weight despite the ease of restriction [35]. These findings underpin the persistent negative impact of the pandemic on pediatric BMI, especially in vulnerable groups such as children with preexisting obesity.

Monitoring these trends and implementing appropriate interventions to address potential long-term consequences on children’s health and well-being is crucial. Our study provides insights into the changes in BMI percentiles among children during and after the COVID-19 lockdown. These findings underscore the importance of promoting healthy lifestyles and supporting children and families during challenging times to mitigate the potential negative impact on weight status. These BMI changes, particularly the weight gain among children who were at a healthy weight at baseline, may lead to long-term health risks. Childhood obesity is strongly linked to a higher risk of diabetes and cardiovascular disease, and these health issues can continue into adulthood [36]. Therefore, early prevention and intervention are essential to reduce these risks.

Although this study is one of the few that have investigated the long-term effects of the COVID-19 lockdown on children’s weight status, some limitations should be considered while interpreting the findings. First, the sample was drawn from a single institution using convenience sampling. This approach may limit the generalizability of the findings to broader populations and could introduce selection bias. Second, one of the limitations of our study is the small sample size of cohort 3. Third, our study has focused solely on changes in body mass during COVID-19. It has not included other potential factors that may influence BMI changes, such as reasons for visits, medical conditions, and screen time, which could affect the interpretation of BMI changes. Furthermore, this study did not control for potential confounding factors like changes in physical activity levels, dietary habits, and socioeconomic status during the pandemic. Additionally, since we are using medical records, we did not account for local determinants such as school policies, climate, culture, and family structure, which could potentially impact the study outcomes. These factors could influence weight status changes and may affect the interpretation of the results. Finally, although using the data of children extracted from medical records may introduce selection bias, it reduces the risk of recall that could be associated with survey-based studies. This underscores the need for further research to validate and extend these results with broader, more representative cohorts and comprehensive adjustment for key confounders.

## 5. Conclusions

The study found that children experienced changes in BMI during and after the COVID-19 lockdown, with younger children and those with obesity showing decreases in BMI during and after the lockdown. In comparison, older children and healthy-weight children had a significant increase in their adjusted BMIs. These findings emphasize the importance of tailored interventions to promote healthy lifestyles and support children, particularly during challenging times like the COVID-19 pandemic. Future research with a larger sample size and primary data that include lifestyle, behavioral, and socioeconomic factors is recommended to better understand the determinants of BMI changes and improve generalizability.

## Figures and Tables

**Table 1:** Characteristics of cohort 1 (N = 934) and their univariate associations with mean changes in age and Sex-adjusted BMI Percentile (during COVID-19 lockdown).

Variable		N (%)	Change in the Adjusted BMI Percentile		
			Mean ± SD	*p*-Value	Effect Size
**Total**		**934 (100)**	**0.06 ± 0.24**		
**Gender**				**0.704 ^a^**	**−0.025**
	Male	508 (54.4)	0.06 ± 0.24		
	Female	426 (45.6)	0.06 ± 0.22		
**Age groups**				**<0.001 ^b^**	**0.032**
	Less than or equal to 6 years	44 (4.7)	−0.13 ± 0.31		
	7–12 years	469 (50.2)	0.08 ± 0.26		
	More than 12 years	421 (45.1)	0.05 ± 0.18		
**The baseline weight status**				**<0.001 ^b^**	**0.060**
	Underweight	166 (17.8)	0.13 ± 0.21		
	Healthy weight	559 (59.9)	0.08 ± 0.25		
	Overweight	98 (10.5)	−0.05 ± 0.19		
	Obese	111 (11.9)	−0.04 ± 0.13		

^a^: *p*-value of independent *t*-test; ^b^: *p*-value of one-way-ANOVA.

**Table 2:** Characteristics of cohort 2 (N = 1129) and their univariate associations with mean changes in age and Sex-adjusted BMI Percentile (after COVID-19 lockdown).

Variable		N (%)	Change in the Adjusted BMI Percentile		
			Mean ± SD	*p*-Value	Effect Size
**Total**		**1129 (100)**	**0.01 ± 0.19**		
**Gender**				**0.887 ^a^**	**0.008**
	Male	583 (51.6)	0.01 ± 0.20		
	Female	546 (48.4)	0.01 ± 0.18		
**Age groups**				**<0.001 ^b^**	**0.015**
	Less than or equals to 6 years	457 (40.5)	−0.02 ± 0.25		
	7–12 years	441 (39.1)	0.03 ± 0.13		
	More than 12 years	231 (20.5)	0.02 ±0.13		
**The baseline Body Mass Index Categories**				**<0.001 ^b^**	**0.039**
	Underweight	151 (13.4)	0.08 ± 0.18		
	Healthy weight	652 (56.8)	0.02 ± 0.21		
	Overweight	136 (12.0)	−0.05 ± 0.15		
	Obese	190 (16.8)	−0.04 ± 0.15		

^a^: *p*-value of independent *t*-test; ^b^: *p*-value of one-way-ANOVA.

**Table 3:** Characteristics of cohort 3 (N = 203) and their univariate associations with the adjusted BMI Percentile during three times series (repeated measures).

Variable		N (%)	The Mean of the Adjusted BMI Percentile ± SD				
			Before COVID-19 Lockdown	During COVID-19 Lockdown	After COVID-19 Lockdown	*df*	*p*-Value
**Total**		**203 (100)**	**0.4 ± 0.36**	**0.53 ± 0.37**	**0.56 ± 0.37**	**2**	<0.001 *
**Gender**						**1**	0.04 *
	Male	110 (54.2)	0.44 ± 0.36	0.54 ± 0.38	0.57 ± 0.38		
	Female	93 (45.8)	0.39 ± 0.36	0.51 ± 0.37	0.55 ± 0.36		
**Age groups**						**2**	0.247 *
	Less than or equals to 6 years	5 (2.5)	0.56 ± 0.45	0.59 ± 0.54	0.48 ± 0.49		
	7–12 years	123 (60.6)	0.39 ± 0.33	0.51 ± 0.54	0.55 ± 0.36		
	More than 12 years	75 (36.9)	0.43 ± 0.39	0.56 ± 0.38	0.59 ± 0.38		
**The baseline Body Mass Index Categories**						**3**	<0.001 *
	Underweight	47 (23.2)	0.01 ± 0.01	0.15 ± 0.22	0.18 ± 0.25		
	Healthy weight	117 (57.6)	0.40 ± 0.26	0.54 ± 0.32	0.59 ± 0.31		
	Overweight	22 (10.8)	0.90 ± 0.03	0.89 ± 0.23	0.88 ± 0.21		
	Obese	17 (8.4)	0.98 ± 0.02	0.98 ± 0.02	0.98 ± 0.02		

* The repeated-measures analysis of variance (ANOVA).

## Data Availability

The data presented in this study are available on request from the corresponding author. The data used in our study were obtained from medical records. Although all identifying information was removed, our Institutional Review Board (IRB) approval includes a commitment that the data will not be published or disseminated publicly. In line with ethical standards, the data were approved for use strictly for scientific research purposes and are scheduled to be deleted after publication. However, should there be a reasonable or warranted request, we are prepared to provide the data in a manner that complies with our ethical and institutional guidelines.
